# Colorenal Fistula in a Patient With Recurrent Urinary Tract Infections and Urinary Urgency: A Case Report

**DOI:** 10.7759/cureus.104446

**Published:** 2026-02-28

**Authors:** Joey E Maksoud, Lauren Peter, Elissa Zakharia, Brady Werth, Philippe Nabbout

**Affiliations:** 1 Urology, University of Kansas Medical Center, Kansas City, USA; 2 Urology, Kansas City University, Kansas City, USA; 3 Surgery, Wichita Surgical Specialists, Wichita, USA; 4 Urology, Wichita Urology Group, Wichita, USA

**Keywords:** colo-renal fistula, recurrent nephrolithiasis, urinary tract infections, urinary urgency, urology department

## Abstract

Colorenal fistula is a rare condition resulting from chronic inflammation, malignancies, diverticulitis, or infection. This case examines a 68-year-old female with a history of urinary urgency, left back pain, and recurrent urinary tract infections. Anteroposterior non-contrast computed tomography scan showed a large stone in the renal pelvis and gas in the collecting system. Left ureteroscopy revealed multiple ureteral polyps. Retrograde pyelogram revealed contrast filling the left colon. Ureteral polyp biopsies were benign, and colonoscopy was negative for malignancy. This case describes the occurrence of a colorenal fistula in a patient with no history of surgery, malignancy, or diverticulitis.

## Introduction

Colorenal (CR) fistulas are pathologic connections between the colon and the kidney with poor incidence documentation in contemporary literature due to rarity. Fistulas are usually manifestations of underlying disease or trauma to the surrounding area, including surgical or radiological interventions. These fistulas are rare because the kidney and the colon are anatomically separated by Gerota's fascia, the retroperitoneal fat, and the peritoneum. CR fistulas may arise from a variety of causes, most frequently including Crohn’s disease, xanthogranulomatous pyelonephritis (a rare chronic form of infection usually secondary to obstruction), malignancy, and staghorn calculi [1]. It is rare to appreciate the development of a CR fistula in patients with only recurrent urinary tract infections (UTIs) and nephrolithiasis. These fistulas most commonly present with urologic symptoms such as pneumaturia (68%), dysuria (64%), recurrent UTIs (32%), and fecaluria (28%) [2]. On the other hand, colonic symptoms are frequently absent [3]. However, CR fistulas manifest with various clinical presentations, delaying diagnosis and increasing morbidity [4].

The most common pathophysiology for the development of a CR fistula is the product of chronic inflammation, tissue necrosis, and infection, resulting in erosion through the renal parenchyma into the adjacent colon [1]. Obstructive uropathy with secondary chronic infection may exacerbate this process as well [4]. These fistulas tend to favor left kidney involvement due to the closer proximity to the descending colon than the right kidney and the ascending colon [3]. Diagnosis requires a multimodal approach, most often consisting of a combination of cross-sectional imaging, primarily computed tomography (CT), and cystoscopy. CT of the abdomen and pelvis with contrast may enhance fistulous connections, reveal abscesses or inflammatory masses, and demonstrate gas movement between the colon and urinary tract [5,6]. However, colonic imaging may also appear normal, increasing the difficulty of diagnosis. In patients with CR fistulas secondary to Crohn’s disease, CT of the abdomen and pelvis has a 52% diagnostic yield. Additionally, cystoscopy has a 74% diagnostic yield for urinary tract fistulas [2]. In cases of high suspicion with unclear imaging, the addition of water-soluble contrast, either orally/rectally or via retrograde bladder filling, may aid in diagnosis [7,8].

Treatment depends on the underlying etiology, presence of complications, and severity of symptoms. In rare cases, conservative management can be used [9]. However, most cases require surgical resection of the inflamed bowel with closure of the renal defect [10,11]. In cases with primary renal etiology, treatment usually involves total nephrectomy [4].

Contemporary literature on CR fistulas is very limited, with no current literature to our knowledge presenting the case of a CR fistula in the absence of malignancy, obstructive uropathy, or iatrogenic causes. We report a rare case of a 68-year-old female without common predisposing factors, including prior surgical history, malignancy, obstructive uropathy, or diverticular disease, presenting with recurrent UTIs, nephrolithiasis, and left flank pain. Imaging combined with operative findings revealed a fistulous connection between the left kidney and the sigmoid colon. This case emphasizes the importance of considering rare complications like CR fistulas in the differential diagnosis of patients with recurrent UTIs and non-staghorn nephrolithiasis that present with atypical imaging findings.

This article was presented as a poster at the Society of Genitourinary Reconstructive Surgeons in September of 2025 in Minneapolis, Minnesota.

## Case presentation

A 68-year-old female patient with no significant oncologic or surgical history presented to an outpatient urology clinic with recurrent UTIs for six months, urinary frequency and urgency, and lower, left-sided back pain. The patient denied hematuria, weight loss, and constitutional symptoms. The patient's previous UTIs have been treated with antibiotics, including ciprofloxacin, Bactrim, and cephalexin. Physical exam revealed left costovertebral angle tenderness without other significant findings. Complete blood count and comprehensive metabolic panel showed no leukocytosis and normal creatinine levels. Inflammatory markers were not obtained. Urinalysis was consistent with signs of infection, showing leukocytosis but no fecaluria or pneumaturia. Urine cultures revealed infection with *Pseudomonas aeruginosa*.

**Figure 1:**
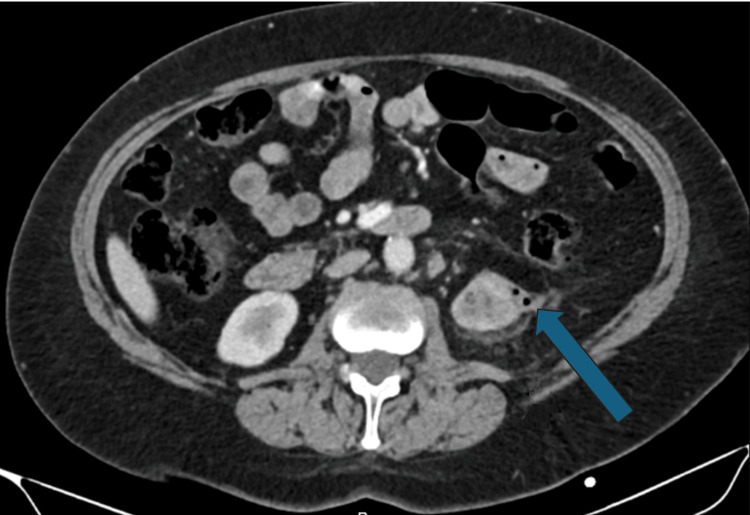
Axial non-contrast CT scan shows gas in the left kidney, indicated by the blue arrow, suggesting the presence of a fistulous connection to the bowel or emphysematous pyelonephritis.

**Figure 2:**
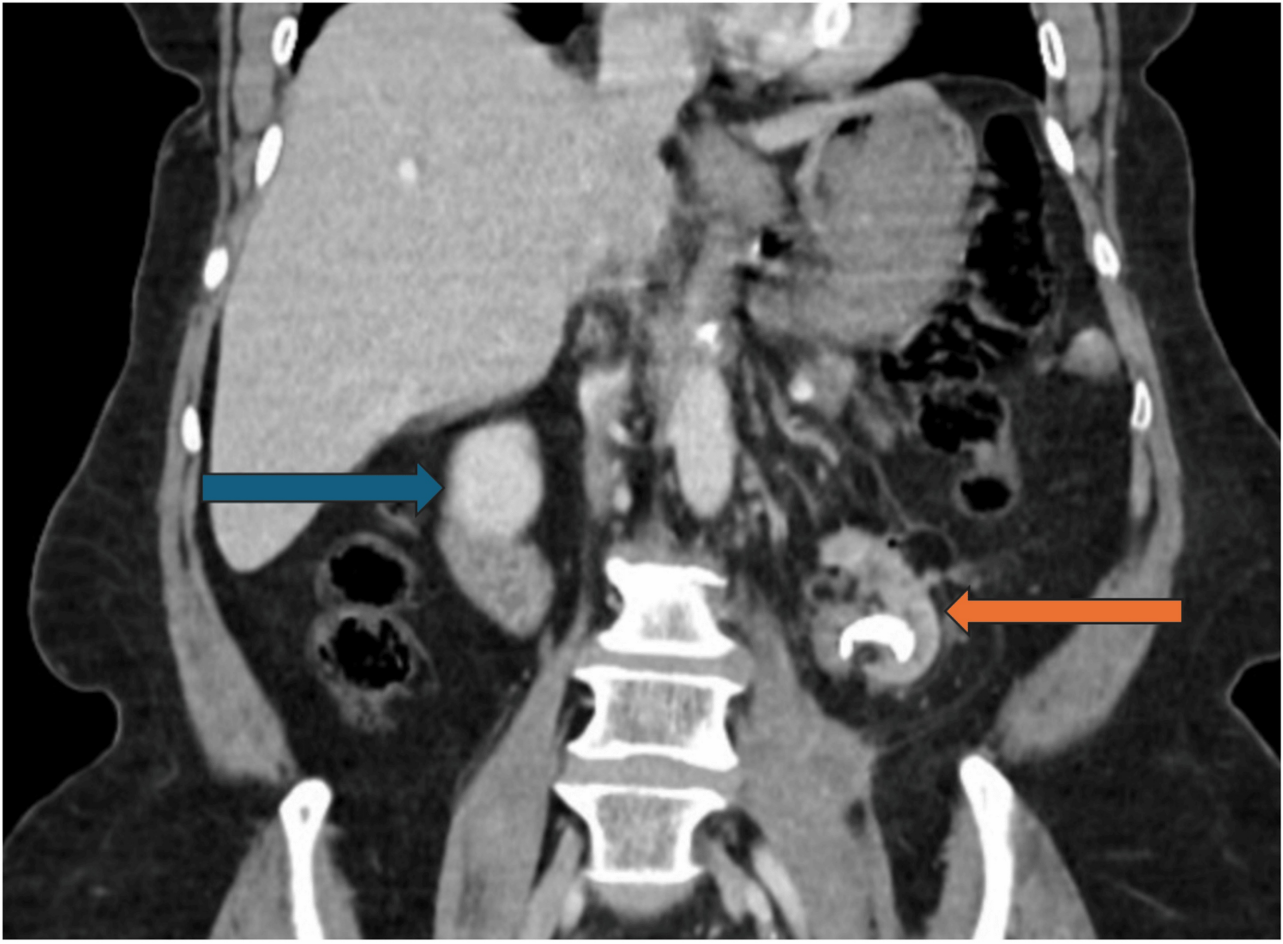
Coronal CT scan showing an atrophic left kidney, indicated by the orange arrow, and a simple cyst in the right kidney, indicated by the blue arrow.

A non-contrast CT scan was completed by the patient's primary care provider prior to the visit, and the patient declined a contrast CT due to cost. A non-contrast CT of the abdomen and pelvis revealed marked atrophy of the left kidney, perinephric fat stranding, mild left hydronephrosis, retroperitoneal lymphadenopathy, a 2.3 cm calculus within the left renal pelvis, and gas within the left collecting system. The results of the CT scan raised suspicion of a fistulous connection between the kidney and bowel (Figure 1). These findings could also suggest emphysematous pyelonephritis. However, the clinical picture did not support a septic background. Moreover, the proximity of the colon on CT further increased suspicion for a fistulous connection. The CT scan also revealed a simple cyst and otherwise normal findings for the right kidney (Figure 2).

**Figure 3:**
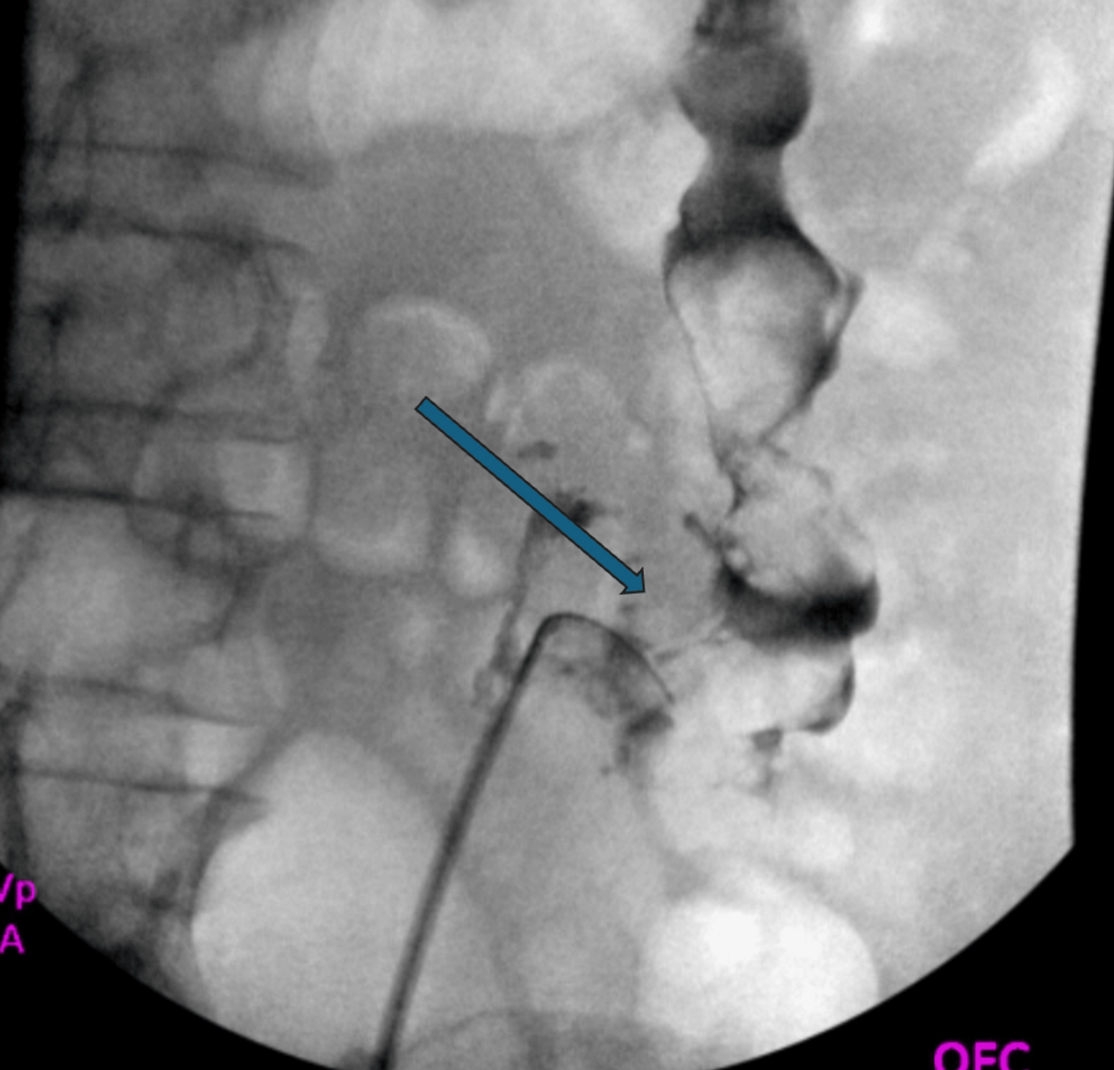
Retrograde pyelogram shows leakage of the left collecting system into the colon, indicated by the blue arrow.

Consequently, the patient underwent cystoscopy with retrograde pyelogram two months later to examine and visualize these findings. A retrograde pyelogram is a form of X-ray imaging that uses contrast dye injected directly into the ureters during cystoscopy to visualize the upper urinary tract. Retrograde pyelogram imaging showed contrast leakage into the sigmoid colon, confirming communication between the kidney and colon and ruling out emphysematous pyelonephritis (Figure 3). In an attempt at further visualization, a flexible ureteroscope was introduced. However, due to chronic inflammation, visualization throughout the left ureter was poor. Mucopurulent material and necrotic debris were appreciated in a severely diseased collecting system with no normal urothelium. The renal calculus and fistulous connection were not visualized on examination. Several inflammatory ureteral polyps were appreciated and biopsied with a zero-tip basket. Histopathological evaluation of these polyps revealed granulation tissue and necrosis with no evidence of malignancy. Lastly, a double-J stent (6 Fr x 24 cm) was placed to decompress the urinary tract. The postoperative period was uncomplicated, with discharge the next day.

Further investigation with colonoscopy was unremarkable, with no evidence of malignancy, diverticulosis, Crohn's disease, or mucosal abnormalities. The patient continued to report symptoms. Due to imaging consistent with findings for a CR fistula, intraoperative findings of mucopurulent material and necrotic debris using ureteroscopy, and the patient’s continued discomfort, the patient underwent a combined open transperitoneal left nephrectomy and sigmoid colectomy with primary anastomosis three months later. A simple nephrectomy was chosen instead of a partial nephrectomy due to significant atrophy appreciated on CT imaging and the potential for further UTI if part of the kidney remains, serving as a nidus for infection.

**Figure 4:**
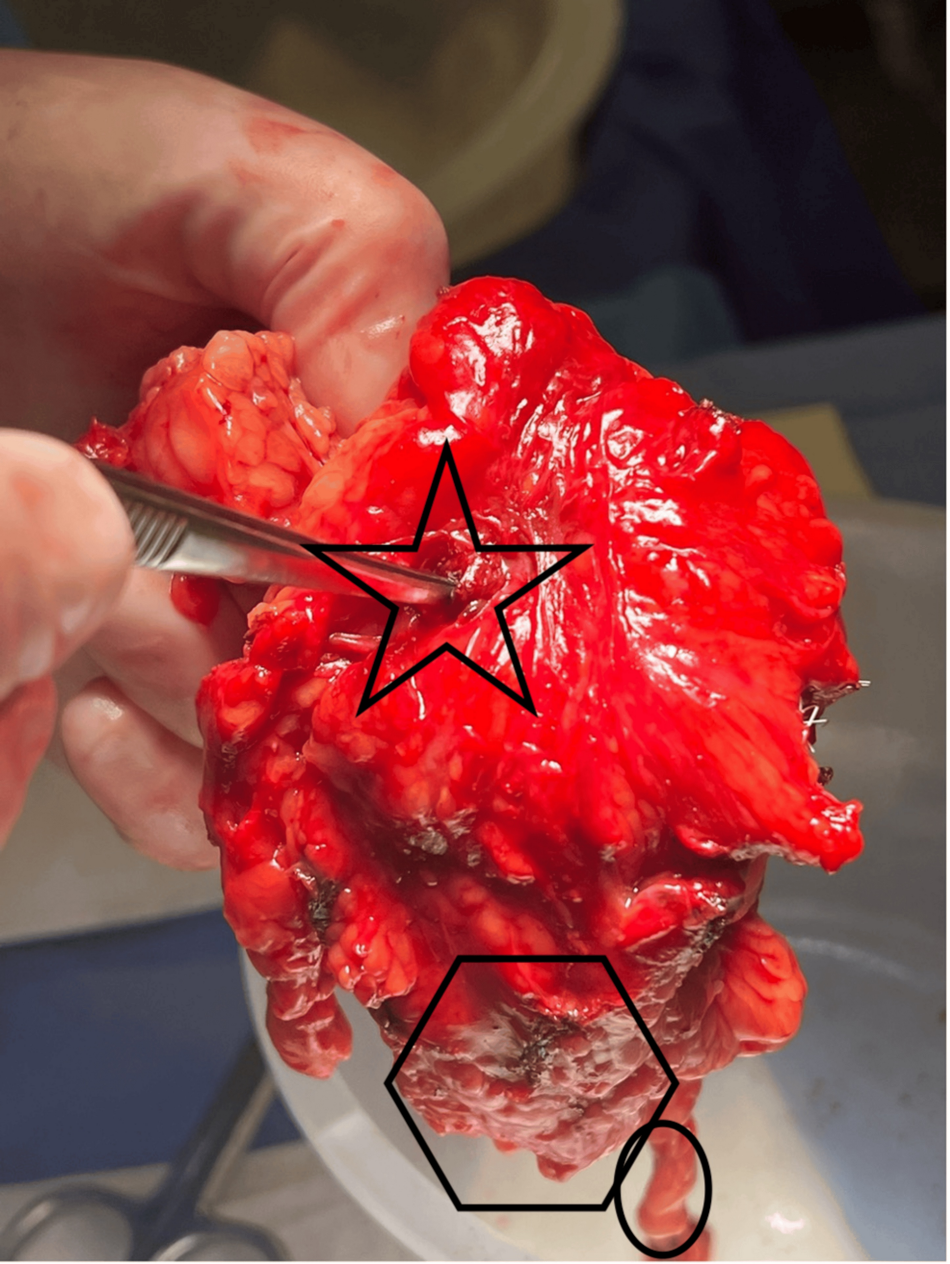
Gross image presents the isolated fistulous sinus tract. The oval outlines the ureter. The star outlines the fistulous tract. The polygon outlines atrophic renal tissue.

**Figure 5:**
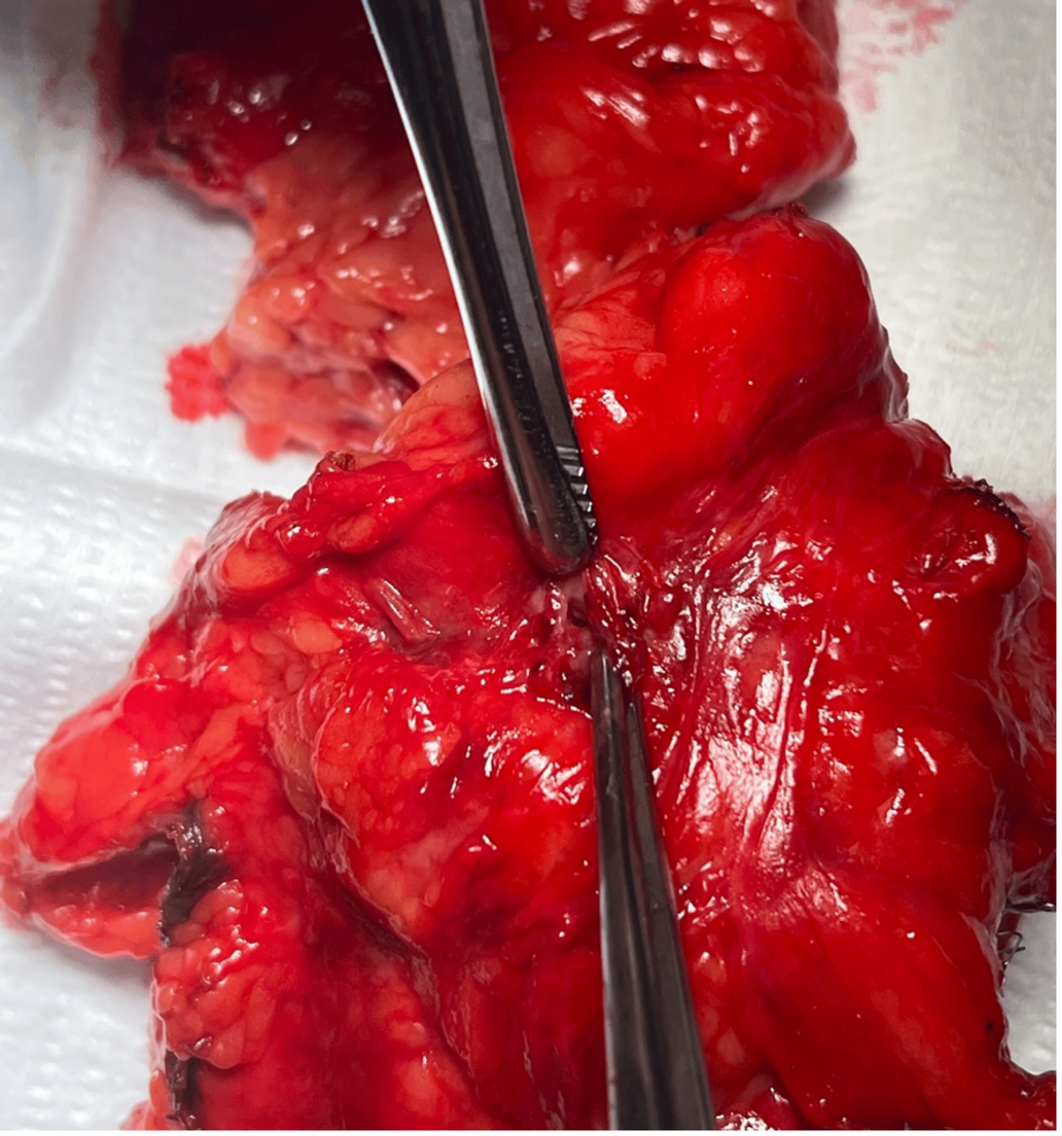
Closer view of the sinus tract, revealing colonic mucosa.

**Figure 6:**
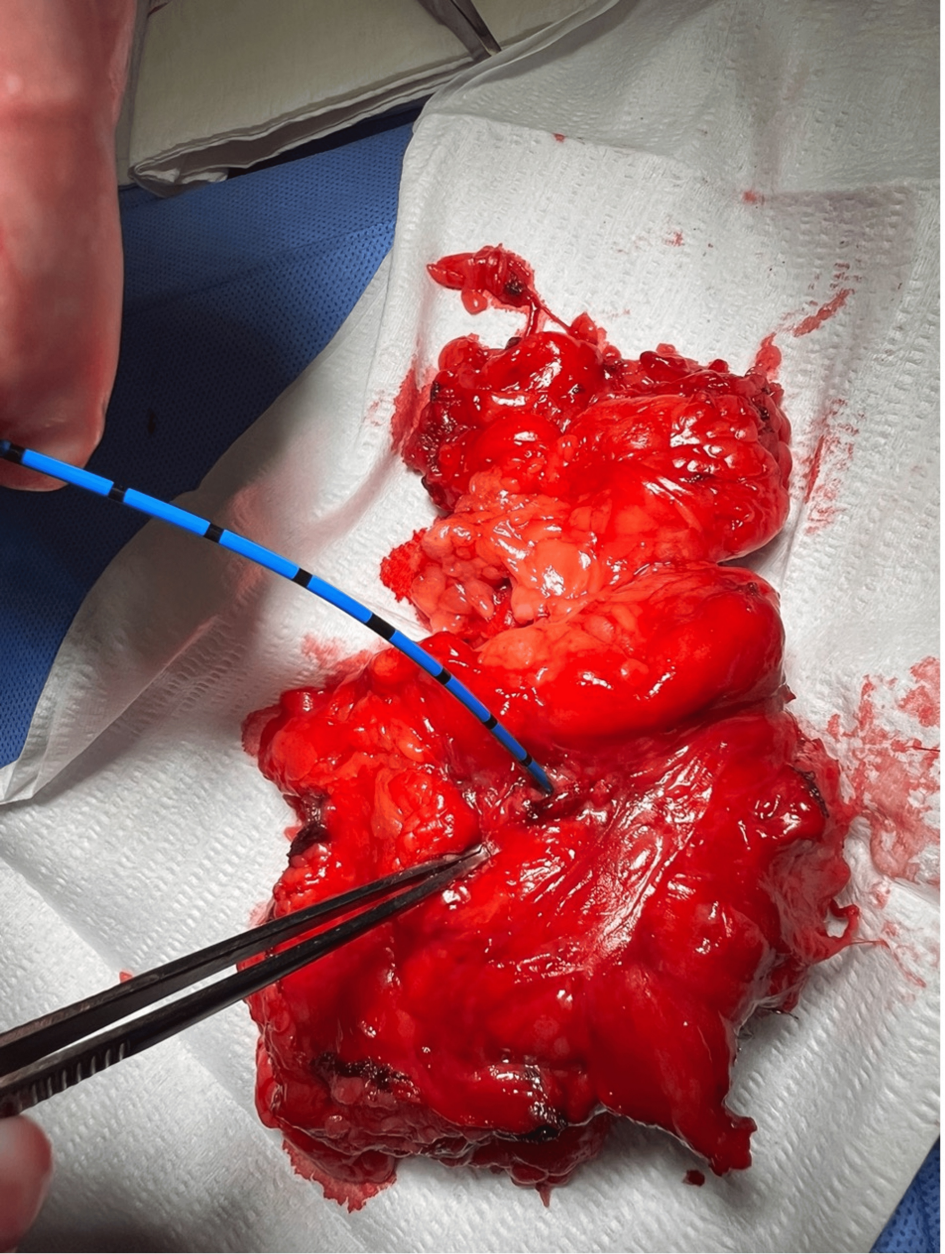
Passage of a ureteral catheter through the fistulous communication into the colon confirms the existence of a fistula.

Gross pathology of the resected left kidney confirmed a CR fistula. Pathology revealed severe atrophy, acute and chronic inflammation, abscess formation, and a 3.0 cm sinus tract stretching from the renal pelvis to the perinephric fat (Figures 4-6). Figure 4 presents the fistulous tract, along with severely atrophied renal tissue. The atrophied renal tissue supports the decision to remove the kidney because tissue this atrophic would not serve a functional role in the urinary tract. Further, if this tissue remained, it would serve as a nidus for further infection to recur. Additionally, a 2.3 cm stone in the left renal pelvis was noted. Pathology of the colonoscopy revealed no malignancy, diverticulosis, or Crohn's disease. Gross pathology of the resected sigmoid colon revealed a 1.5 cm area of luminal stricture with fibrosis and hemorrhage, confirming the presence of a fistulous communication.

There were no complications postoperatively, and the patient remained infection-free in the postoperative period. The patient was discharged on postoperative day three. The patient had resolution of urinary symptoms and no recurrence of back pain at follow-up. The patient followed up in the clinic three months later with resolved symptoms. They were then discharged from the clinic. The patient's primary care provider agreed to refer the patient back to this clinic if symptoms ever recurred. The patient has not been referred back after over a year.

This study presents the case of a 68-year-old female presenting with chronic urinary inflammation secondary to recurrent UTIs. In the absence of common etiologies like malignancy, diverticulitis, or obstructive calculi, this case presents a CR fistula. These fistulas are very rare due to anatomical boundaries and are poorly documented in modern literature, emphasizing the importance of this study. Table 1 presents the critical details of the case to enhance clarity of case presentation.

**Table 1:** A brief summary table of patient presentation, clinical red flags, imaging/endoscopic findings, management, pathology, and outcome to enhance clarity of case presentation.

Category	Findings & details
Patient presentation	A 68-year-old female with a six-month history of recurrent UTIs, urinary frequency/urgency, and left-sided back pain. No history of malignancy, Crohn’s, obstructive uropathy, or prior abdominal surgery.
Clinical "red flags"	Left costovertebral angle tenderness; urine culture positive for *Pseudomonas aeruginosa*; absence of typical symptoms like fecaluria or pneumaturia.
Initial imaging (non-contrast CT)	Atrophic left kidney with gas in the collecting system, perinephric fat stranding, mild hydronephrosis, and a 2.3 cm renal pelvis calculus.
Confirmatory diagnostics	Retrograde pyelogram showed contrast leakage from the renal collecting system into the sigmoid colon.
Endoscopic findings	Ureteroscopy revealed mucopurulent material, necrotic debris, and inflammatory ureteral polyps, though the fistula itself was not directly seen.
Surgical management	Combined open transperitoneal left simple nephrectomy and sigmoid colectomy with primary anastomosis.
Pathological confirmation	Confirmed a 3.0 cm fistulous sinus tract, severe renal atrophy, chronic inflammation, and renal abscess. The sigmoid colon showed fibrosis and hemorrhage at the communication site.
Outcome	Resolution of all urinary symptoms and back pain; patient remained infection-free at one-year follow-up.

## Discussion

CR fistula is a rare and frequently missed diagnosis that may result from conditions of chronic inflammation, such as chronic pyelonephritis, malignancy, diverticulitis, and Crohn’s disease. Well-established etiologies include iatrogenic causes from surgical intervention. Published literature on the development of these fistulas in the absence of the named etiologies is limited. In this case, we describe the presentation of a CR fistula in a patient with no significant surgical history, diverticular disease, malignancy, or Crohn’s disease. The absence of these conditions suggests that chronic infection associated with recurrent UTIs in the setting of an obstructing renal calculus may have contributed to fistula formation, although the etiology remains multifactorial and cannot be definitively established.

This case demonstrates the varied and often subtle presentation of these fistulas: a patient with recurrent UTIs and left-sided back pain without any pneumaturia, fecaluria, or gastrointestinal symptoms. This patient’s nonspecific symptoms, combined with her normal colonoscopy, highlight the diagnostic challenge of CR fistulas. CT scan without contrast of the abdomen and pelvis significantly increased our clinical suspicion, revealing a left renal pelvic stone, renal atrophy, and intrarenal gas. Non-contrast CT of the abdomen and pelvis significantly increased clinical suspicion, suggesting emphysematous pyelonephritis or the possibility of entero-urinary communication in the setting of chronic inflammation, but further diagnostic clarification was required, including retrograde pyelography, due to the limited sensitivity of non-contrast CT for direct fistula visualization. Retrograde pyelography revealed contrast extravasation in the sigmoid colon, confirming the suspected diagnosis of a CR fistula.

Upon endoscopic evaluation, we discovered a grossly diseased left renal collecting system with extensive mucopurulent debris, mucus, and benign ureteral polyps. Although no fistulous connection was identified, the mucus and debris further supported our suspicion that they had a gastrointestinal source. Despite the severity of the diseased kidney, no stone was visualized intraoperatively. This may have been due to the degree of anatomical obscuration secondary to inflammation and chronic disease, as well as a heavy debris burden leading to poor visualization.

CR fistulas are usually treated with surgical resection of the affected colon and renal closure [10,11]. However, due to extensive renal parenchymal destruction, ongoing infection, and poor functional potential of the affected kidney, definitive management with nephrectomy and segmental colectomy was pursued, consistent with reported management strategies for advanced CR fistulas [4]. This patient underwent a successful open transperitoneal left simple nephrectomy and sigmoid colectomy with a primary anastomosis. Pathology confirmed the presence of a 3.0 cm fistulous sinus tract, renal abscess formation, and chronic pyelonephritis, with no evidence of malignancy. Further colonoscopy revealed no diverticular, chronic inflammatory, inflammatory bowel disease, or malignant disease of the colon. The suspected organ of origin was the kidney due to a history of chronic inflammation secondary to infection and nephrolithiasis, although direct causality remains unclear.

This case adds to the limited literature on CR fistulas by describing fistula formation in the absence of malignancy, diverticulitis, Crohn’s disease, or obstructive/staghorn calculi and in the setting of recurrent UTIs with non-staghorn nephrolithiasis and normal colonoscopic findings. It outlines this development specifically in patients with recurrent UTIs and atypical renal imaging. In a diagnosis that commonly presents with subtle and varied features and for which imaging alone has limited diagnostic capability, clinical suspicion is essential. While the combination of contrast imaging and cystoscopy is the cornerstone for diagnosis, a multimodal approach with maintained clinical suspicion is required. In select cases, conservative management may still be appropriate [9]. However, this patient had ongoing recurrent infection and continued symptoms of back pain in the setting of significant renal damage, making her a strong candidate for surgical intervention. Literature on outcomes of nephrectomy with resection in these patients is very limited. However, in patients with Crohn’s disease or xanthogranulomatous pyelonephritis, surgical intervention has produced favorable outcomes [3,12]. This case supports these findings. The patient had no postoperative complications, with resolution of symptoms and no recurrent infection in the postoperative period.

## Conclusions

This case report underscores the importance of maintaining a high level of suspicion for CR fistulas in patients with chronic inflammation. These patients may suffer from persistent or recurrent UTIs, chronic nephrolithiasis, or abnormal renal imaging. Despite the absence of malignancy, obstructive uropathy, Crohn's disease, or diverticulitis and the absence of classic symptoms such as pneumaturia or fecaluria, imaging and endoscopic findings suggested a fistulous connection, which was confirmed by pathologic assessment of the removed kidney. Fortunately, early diagnosis and prompt surgical intervention prevented further complications and resolved the patient's symptoms. This case adds to the currently limited literature on CR fistulas in the absence of malignancy, Crohn’s disease, staghorn calculi, and xanthogranulomatous pyelonephritis and suggests chronic infection as a potential etiology of the fistulous connection.
